# Nonlinear Ultrasonic Characterization of Processing Defects in Wire Arc Additive Manufacturing 316L Stainless Steel

**DOI:** 10.3390/ma18040805

**Published:** 2025-02-12

**Authors:** Pengfei Wang, Jia Zeng, Dong Lou, Wenjian Zheng, Sanlong Zheng, Bingbing Chen, Zengliang Gao

**Affiliations:** 1 School of Mechanical Engineering, Zhejiang University of Technology, Hangzhou 310023, China; pfwang@zjut.edu.cn (P.W.);; 2 Engineering Research Center of Process Equipment and Re-Manufacturing, Ministry of Education, Zhejiang University of Technology, Hangzhou 310023, China; 3 School of Mechanical and Aerospace Engineering, Nanyang Technological University, Singapore 639798, Singapore

**Keywords:** wire arc additive manufacturing, processing defects, porosity, nonlinear ultrasonic

## Abstract

Wire Arc Additive Manufacturing (WAAM) 316L stainless steel unavoidably introduces defects such as porosity, oxide inclusions, and lack of fusion due to the inherent characteristics of the process. These defects can significantly affect the mechanical properties and service reliability of the material. This study focused on evaluating the defects in WAAM 316L stainless steel by nonlinear ultrasonic testing based on Lamb waves. The effects of FCAW (flux cored arc welding) parameters, including shielding gases (98% Ar + 2% O_2_ and 100% CO_2_) and welding speeds (20, 30, and 40 cm/min), on the columnar grain, porosity, and defect types were systematically analyzed. The formed specimens were then subjected to nonlinear ultrasonic testing, and the results showed that the ultrasonic nonlinear parameters exhibited high sensitivity to changes in porosity. This suggests that nonlinear ultrasonic testing can effectively assess processing defects in WAAM 316L stainless steel. The findings provide valuable insights for optimizing the WAAM process and improving the reliability of additive manufacturing components.

## 1. Introduction

Compared to conventional manufacturing techniques, additive manufacturing (AM) has been shown to possess inherent advantages in the fabrication of complex structural components, and has been adopted in a wide variety of industrial applications, including those in the aerospace, medical, and automotive sectors [1]. Among the various AM methods, Wire Arc Additive Manufacturing (WAAM) is a Directed Energy Deposition (DED) technology that deposits raw materials directly into the molten pool generated by the energy source. In comparison with other AM techniques, such as laser and electron beam-based methods, WAAM offers several advantages, including lower equipment costs, higher material utilization efficiency, superior production efficiency, and excellent weld layer density [2,3]. These characteristics render WAAM particularly suitable for fabricating large-scale structures.

However, WAAM is susceptible to various influencing factors during the fabrication process, which results in intricate phase transformations, microstructural changes, uneven residual stresses, deformations, and the accumulation of defects, such as micropores. These micro-defects can act as stress concentration sites during service, potentially leading to the initiation of microcracks in defect-rich regions. This compromises the safety, stability, and reliability of components over extended periods of use. Consequently, accurately assessing the defect characterization and mechanical properties of WAAM components becomes critically important.

Microporosity is a prevalent defect in WAAM, with the capacity to markedly compromise the material’s mechanical properties. Defects such as residual stress, surface roughness, cracking, and delamination can be mitigated by modifying the manufacturing process or by applying post-processing heat treatments. However, microporosity in WAAM materials remains a persistent challenge, with the density and distribution of these micro-defects proving critical factors in determining the performance of the material [4,5,6]. Nonlinear ultrasonic technology has emerged as a significant advancement in the detection of small and early-stage damage defects. A plethora of studies have been conducted by researchers on nonlinear ultrasonic testing technology, encompassing various domains including early-stage mechanical property degradation, non-metallic inclusion detection, and fatigue crack propagation [7,8,9].

In their study of the Ti-6Al-4V alloy in WAAM, Zhang [10] established that the presence of micropores does not affect the tensile strength, but significantly reduces both ductility and fatigue strength. Utilizing fracture mechanics methods and the Murakami stress intensity factor equation for micropores, they developed a fatigue strength prediction method that considers the size, location, and distribution of micropore defects. In a similar vein, Voisin et al. [11] discovered that strain failure in materials is highly sensitive to micropores, with their presence even at below 1% porosity having a significant effect on material elongation. This is due to the tendency of micropores to expand and merge under stress. Gong et al. [12] prepared Ti-6Al-4V materials with different porosities using Selective Laser Melting (SLM) and Electron Beam Melting (EBM). Their study found that when the porosity is below 1%, there is little effect on the material’s mechanical properties. However, when the porosity increases to 5%, a significant decrease in tensile strength and fatigue performance is observed. The study also established that when large unmelted defects (greater than 100 μm) and large macropores (greater than 300 μm) are present within the material, porosity becomes less significant in determining the material’s mechanical properties. In such cases, these macroscopic defects have a substantial impact on all mechanical properties of the material.

In the field of damage detection for additive manufacturing materials, Acevedo, Honarvar, and Prevorovsky et al. [13,14,15] conducted a review of the application of ultrasonic testing technology in additive manufacturing products. At present, the majority of ultrasonic testing in additive manufacturing products is conducted offline, although some studies have utilized in situ ultrasonic testing. These scholars discussed in detail the application of various ultrasonic testing methods for damage detection in additive manufacturing products. In a separate study, Liu [16] introduced a new sideband peak calculation technique for in situ porosity monitoring in 316L stainless steel produced by additive manufacturing. By monitoring the porosity of the samples, it was found that the proposed nonlinear technique is highly sensitive to porosity, making it more suitable for in situ porosity monitoring. Zamen [17] applied nonlinear ultrasound for non-destructive testing of manufacturing defects in additive manufacturing. This approach enabled the detection of defects that were difficult to identify using conventional methods, and their characterization was facilitated by quantitative damage-sensitive parameters. Wang [18,19] utilized a nonlinear ultrasonic technique to obtain the relationship curves between ultrasonic nonlinear parameters and fatigue cycles under three distinct loading conditions for the KMN-I material. The findings revealed that with an increase in fatigue life, the microstructure of the specimen underwent gradual deterioration (with defects such as pores and pits appearing), and subsequent alterations in nonlinear parameters. This study underscores the potential of ultrasonic nonlinear parameters in reflecting the microstructural defects and damage extent of materials. In summary, the utilization of nonlinear ultrasonic testing technology for the characterization of the internal microstructural defects and damage states of WAAM 316L represents a highly valuable research topic. This approach can provide a theoretical foundation and experimental basis for the nondestructive detection and online monitoring of early damage in high-strength steels fabricated by WAAM.

## 2. Materials and Methods

### 2.1. Experimental Materials

**Figure 1:**
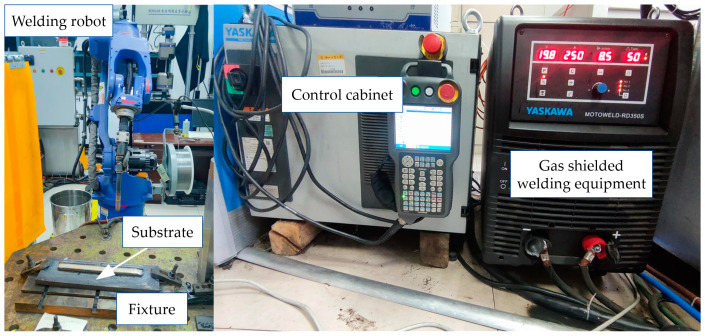
The experimental platform.

In this study, the wire was 316L stainless steel flux-cored wire with a diameter of 1.2 mm, which was produced by Jinqiao Welding Materials Group Co., Ltd. (Tianjin, China). The model of flux-cored wire is E316LTI-1 in AWS. The main chemical composition of 316L stainless steel flux-cored wire is shown in Table 1. The experimental platform mainly consisted of a Yaskawa NX100 six-axis welding robot, a fusion electrode gas shielded welding equipment, a wire feeder, and a shielding gas delivery system, as shown in Figure 1. To ensure the quality of the forming process, a ISO E235B steel plate with dimensions of 400 × 200 × 16 mm was selected as the substrate. As high internal stresses can be generated during the forming process, a fixture was used to hold the substrate in place to prevent warping and deformation. In addition, a laser pyrometer (DL333600 from Deli Tools Co., Ltd., Ningbo, China) was used to monitor the surface temperature of the workpiece to ensure proper control of the interlayer temperature.

The WAAM process is a complex forming technique with key process parameters including welding current, wire feed speed, scanning speed and shielding gas type. Among them, welding current and wire feed speed are interrelated parameters, and their proper coordination plays a significant role in the forming result. The aim of this paper was to study materials with different forming defects, so the welding speed and shielding gas type were chosen as the relevant variables, as shown in Table 2. The remaining FCAW (MAG—136 according to ISO 4063 [20]) parameters included a wire feed speed of 8.5 m/min, a wire extension of 15 mm, a distance from the wire tip to the workpiece of 5 mm, a shielding gas flow rate of 20 L/min, and a welding interval between layers of 3 min. Under these parameters, the experimental current was 140A, the weld width was 8–10 mm, and the Z-height of each layer was about 1.5 mm. The length of the seam was 250 mm for the V20 and V40 plates, and the length of the seam was 390 mm for the V30 plate. Subsequent to the welding of each layer, the processing surface was cleared of slag residue.

**Figure 2:**
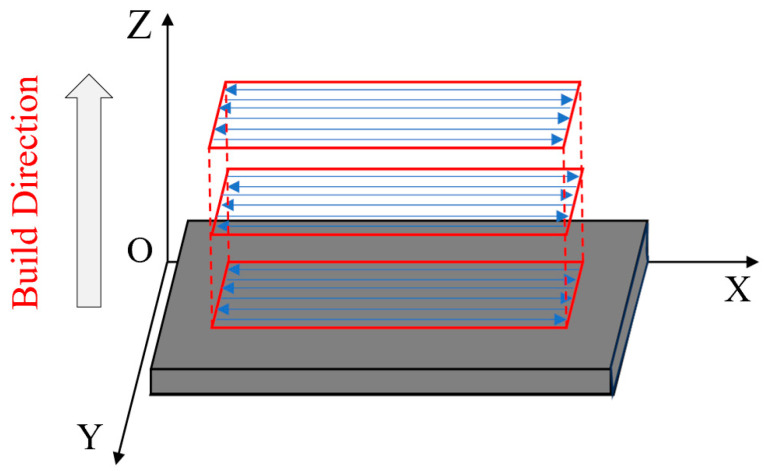
Schematic diagram of the multi-pass and multi-layer deposition directions.

**Figure 3:**
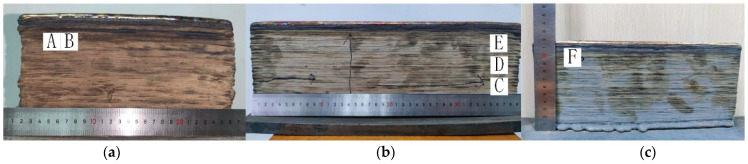
WAAM 316L plates. (**a**) V20; (**b**) V30; (**c**) V40. The letters A to F indicate the sampling positions for subsequent metallographic tests.

In the WAAM process, the initiation of the arc leads to instability in the metal melting process, resulting in excessive material deposition. If each layer is deposited in the same direction, significant height differences occur at both ends, which has a detrimental effect on arc stability and forming performance. Therefore, in this study, a reciprocal deposition approach was used for additive manufacturing, and the schematic diagram of the multi-pass and multi-layer deposition directions used is shown in Figure 2. The plates after WAAM processing are shown in Figure 3.

### 2.2. Experimental Methods

#### 2.2.1. Microstructure Observation

The metallographic organization of WAAM 316L stainless steel was observed using an Olympus BX53M microscope (Tokyo, Japan). Initially, the specimens underwent mechanical grinding and polishing to achieve a smooth surface. Subsequently, chemical etching was performed using aqua regia for a duration of 3–5 s. In order to study the effect of welding speed and forming height on microstructure, three plates processed at different welding speeds were selected for sampling, as shown in Figure 3. Specimens B, E, and F were taken from plates with welding speeds of 20, 30, and 40, respectively, and were located at the same height. Specimens C, D, and E were all taken from plates with a welding speed of 30, and all three were gradually increased along the build direction.

**Figure 4:**
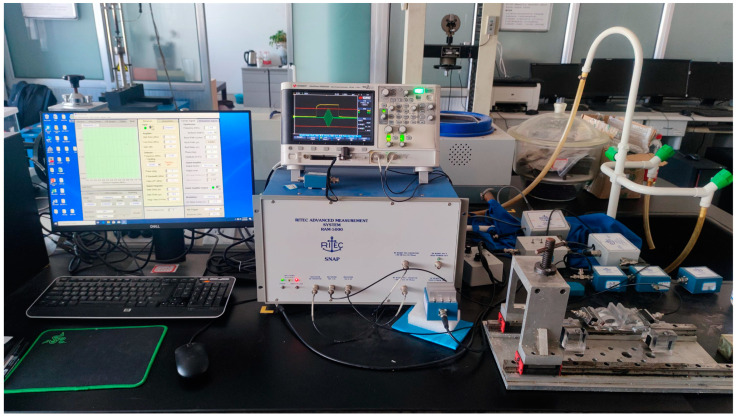
Nonlinear ultrasonic testing system.

A Phenom XL scanning electron microscope (see Figure 2, Figure 3 and Figure 4) was utilized to examine internal defects in arc additively manufactured 316L stainless steel. This was followed by an analysis of porosity.

#### 2.2.2. Nonlinear Ultrasonic Detection

It is a well-established fact that higher-order harmonic signals are typically significantly weaker than fundamental signals. In order to obtain distinct higher-order harmonic signals, the testing system must meet the following requirements: firstly, the energy of the excited ultrasonic waves must be sufficiently high to ensure the quality and intensity of the higher-order harmonic signals. Secondly, it is imperative to minimize interference from noise signals generated by the system. Noise may originate from electronic equipment or environmental factors. To reduce the impact of noise, signal processing methods with high signal-to-noise ratios should be employed, and environmental noise interference should be mitigated. Finally, it is essential to ensure that the received time-domain signals have adequate resolution. The resolution is contingent upon the system’s sampling rate and temporal resolution. Higher sampling rates provide better frequency resolution, allowing for more accurate differentiation between fundamental and second harmonic signals.

The RAM-5000 SNAP high-energy ultrasonic system is distinguished by its high energy, high resolution, and excellent reliability, rendering it well-suited to meet the aforementioned requirements. Furthermore, the system is equipped with specialized software for parameter configuration and signal processing. In this study, a nonlinear ultrasonic testing system based on Lamb waves was established using the RAM-5000 SNAP system, including a computer, the RAM-5000 SNAP system, 50 Ω impedance, a 2.25 MHz low-pass filter, an angled transmitter, an angled receiver, a signal amplifier, and an oscilloscope, as illustrated in Figure 4. First, the RAM-5000 SNAP high-energy ultrasonic system was used to generate the ultrasonic signal, which subsequently passed through a 50 Ω resistor for impedance matching. This step was essential to prevent signal reflection during transmission and to ensure efficient signal delivery. Subsequently, the signal was transmitted through a 2.25 MHz low-pass filter to the excitation transducer. The signal was then transmitted into the specimen by the coupled wedge, where it propagated along the specimen. The signal received by the receiving transducer was amplified using a signal amplifier and then sent to the oscilloscope for real-time monitoring.

There is a nonlinear term in the wave equation of the sound wave propagating in the medium. Material performance degradation is always accompanied by some form of material nonlinear mechanical behavior, which causes the generation of nonlinear harmonics of ultrasonic propagation. These high-order harmonics are very sensitive to damage in materials or structures in the early stage.

Due to the nonlinearity of the solid medium, the stress–strain relationship exhibits nonlinear characteristics, which can be expressed as:

(1) $$\sigma =E\varepsilon \left(1+\frac{\beta \varepsilon }{2}+\frac{\delta \varepsilon^{2}}{6}\right)$$

where *σ* is the stress, *ε* is the strain, *E* is the Young’s modulus, and *β* and *δ* are the parameters describing the degree of material nonlinearity, namely, the second-order nonlinear parameter and the third-order nonlinear parameter. Due to the nonlinearity of the solid medium, the ultrasonic and the solid medium have a nonlinear interaction, thus producing higher harmonics. At this time, the one-dimensional longitudinal wave nonlinear wave equation in the solid medium can be expressed as:

(2) $$\rho \frac{\partial^{2}u}{\partial t^{2}}=\frac{\partial \sigma }{\partial x}$$

(3) $$\frac{\partial^{2}u}{\partial t^{2}}=c^{2}\left(1-\beta \frac{\partial u}{\partial x}\right)\frac{\partial^{2}u}{\partial x^{2}}$$

where $c=\sqrt{E/\rho }$ represents the longitudinal wave velocity, *ρ* is the medium density, *u* is the displacement, and *x* is the longitudinal wave propagation distance. Using the second-order perturbation method, the solution of Equation (2) can be obtained as *u* = *u*_0_ + *u*_1_, where *u*_0_ is the solution when *β* = 0, and *u*_1_ is the first-order perturbation solution. Through the iterative process, the specific expression of Equation (3) can be obtained as:

(4) $$u\left(x,t\right)=A_{1}\sin\left(kx-\omega t\right)+\frac{{A_{1}}^{2}k^{2}\beta x}{8}\cos2\left(kx-\omega t\right)$$

where *A*_1_ is the amplitude of the fundamental frequency, *k* is the wave number (*k* = 2*π*/*λ*, *λ* is the wavelength), and *ω* is the angular frequency. The amplitude *A*_2_ of the second harmonic wave propagating in the material is $\frac{{A_{1}}^{2}k^{2}\beta x}{8}$.

Therefore, the ultrasonic nonlinear parameter can be obtained by measuring the amplitude of the ultrasonic fundamental frequency signal and the higher harmonic signal, and the second-order nonlinear parameter *β* can be expressed by Equation (5):

(5) $$\beta =\frac{8}{k^{2}x}\frac{A_{2}}{{A_{1}}^{2}}$$

In the course of the experiment, the transducer frequency and propagation distance were maintained as constant, i.e., *k* and *x* were constants, under the condition that the nonlinear parameter *β* was proportional to $A_{2}/A_{1}^{2}$. Consequently, the relative nonlinear parameter *β’* was conventionally used in nonlinear ultrasonic detection, and it is abbreviated as *β* in the manuscript, that is, $\beta =A_{2}/A_{1}^{2}$. Furthermore, the utilization of a normalized data treatment in our subsequent analyses served to eliminate the effects of *k* and *x* on the nonlinear parameter.

**Figure 5:**
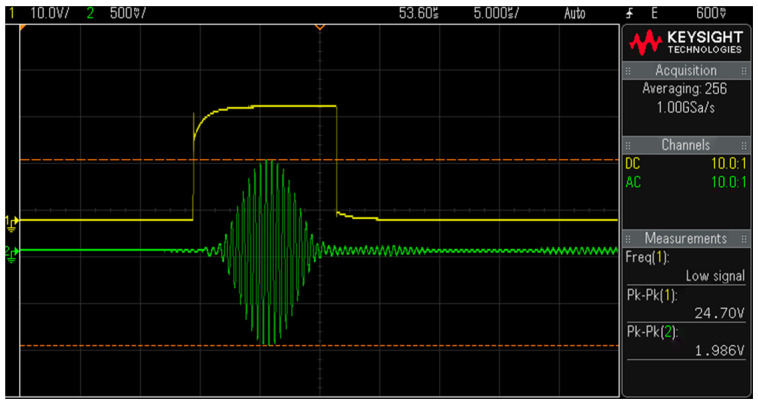
Time-domain signal.

Figure 5 shows the time domain signal on the oscilloscope, where the green curve is the signal received by the receiving probe, and the yellow curve is the gate signal for Fourier integration of the received signal. The time-domain signal was then subjected to a Fast Fourier Transform or a Short Time Fourier Transform (depending on the purity of the received signal) to obtain a frequency-domain signal. Then, we obtained the maximum amplitude of the fundamental wave and second harmonic wave. Subsequently, the nonlinear parameter could be calculated using the formula $\beta =A_{2}/A_{1}^{2}$.

## 3. Results and Discussion

### 3.1. Characteristics of Processing Defects

The grain morphology of 316L stainless steel fabricated by WAAM is closely related to the cooling and heat accumulation mechanisms. In the vicinity of the substrate, the rapid cooling rate promotes the formation of vertically grown columnar dendrites. As the deposition height increases, heat accumulates within the material, leading to continuous grain growth and an increase in dendrite spacing. However, in the intermediate regions of the deposited layers, the grain size and dendrite spacing remain relatively stable. With further increases in deposition height, however, the dissipation of heat towards the lower regions and the substrate becomes increasingly difficult. The accumulated heat, in combination with newly absorbed thermal energy, leads to continued grain growth, larger dendrite spacing, and the enhanced development of secondary dendrites.

**Figure 6:**
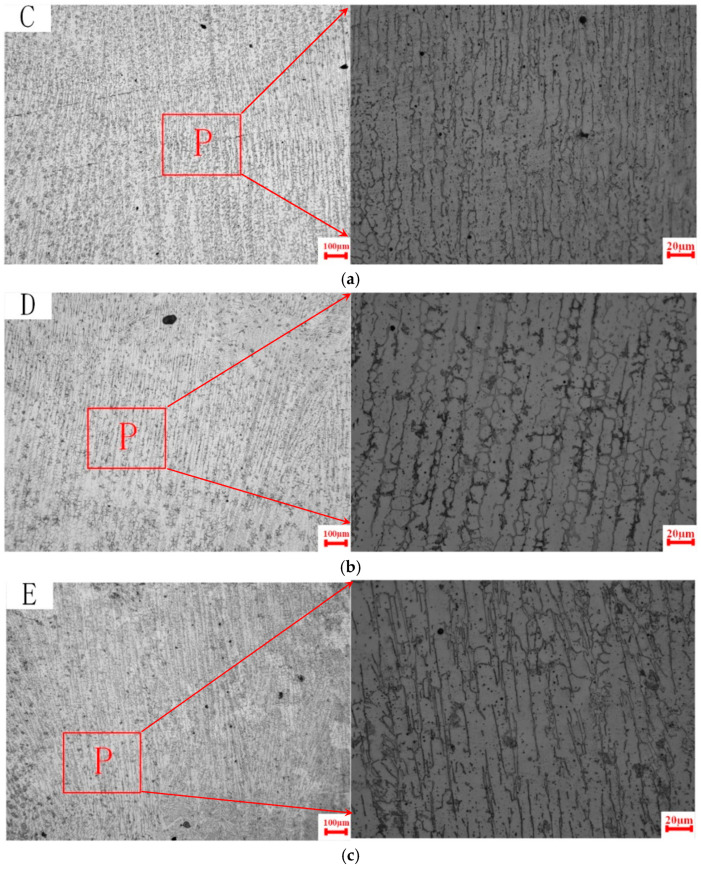
Microstructure of V30 specimens. (**a**) Point C, the bottom; (**b**) point D, the middle; and (**c**) point E, the top.

As illustrated in Figure 6, the vertical cross-sectional microstructure corresponding to positions C, D, and E in Figure 3 is presented under the V30 processing parameters. The right side of the figure shows magnified views of the P region on the left. The microstructure of the cross-section along the deposition direction is predominantly characterized by oriented columnar grains. In the lower region, the columnar dendrite spacing is approximately 7.14 µm, with a small number of secondary dendrites observed. In the middle region, the columnar dendrite spacing increases to approximately 14.81 µm, and the number of secondary dendrites rises, although their morphology is less pronounced. In the upper region, the columnar dendrite spacing increases further to approximately 17.6 µm, and secondary dendrites become clearly visible. These observations indicate that during the gradual accumulation of layers, the columnar grains near the substrate in the lower region are smaller and more compact. The dendrite spacing increases with the number of deposited layers, accompanied by a corresponding increase in the number of secondary dendrites.

**Figure 7:**
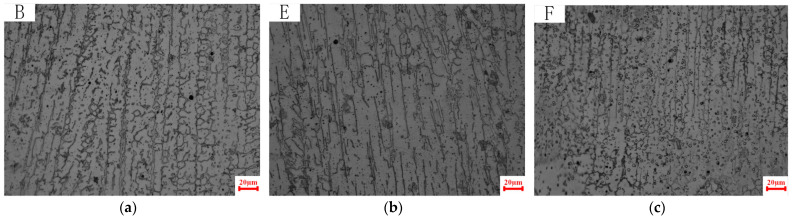
Microstructure at different welding speeds. (**a**) Point B, V20; (**b**) point E, V30; and (**c**) point F, V40.

As illustrated in Figure 7, the microstructures corresponding to positions B, E, and F in Figure 3 are depicted at different scanning speeds at the same height. The columnar dendrite spacing was measured to be approximately 20.77 µm under the V20 parameters; approximately 17.6 µm under the V30 parameters; and approximately 12.04 µm under the V40 parameters. It is evident that the size of the columnar dendrite spacing is primarily influenced by the cooling rate. Increases in welding speed are associated with a rise in the temperature gradient, leading to a continuous decrease in columnar dendrite spacing.

**Figure 8:**
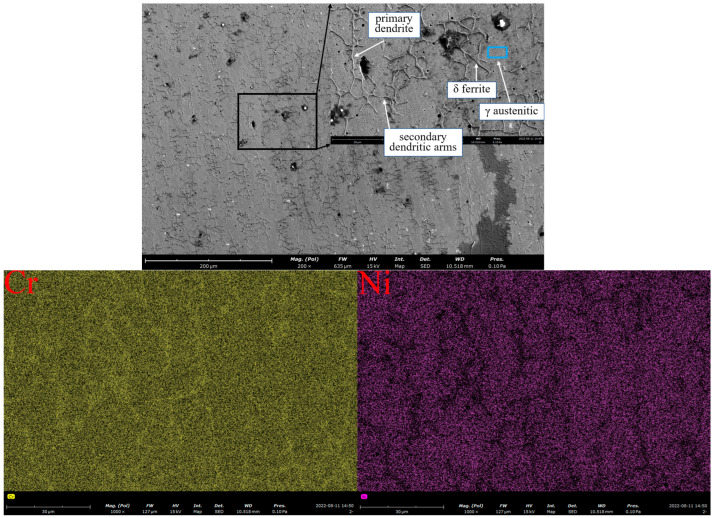
Metallography and element distribution of V20 specimen using SEM.

As illustrated in Figure 8, the WAAM 316L stainless steel comprises a matrix phase of austenite *γ* and a precipitation phase of ferrite *δ*. Among these elements, Cr and Mo are enriched in the ferrite, while Ni is depleted. In austenitic stainless steel, the occurrence of localized element diffusion phenomena is evident. Specifically, ferrite-forming elements, such as Cr, exhibit diffusion from austenite to ferrite, while the austenite-forming elements, including Ni, demonstrate the opposite behavior. This phenomenon is attributed to the short-range diffusion mechanism. EDS spectroscopy was performed on the matrix and precipitation phases of the V20 specimen to analyze the composition and proportion of each element in the matrix and precipitation phases. The specific compositions are shown in Table 3.

**Figure 9:**
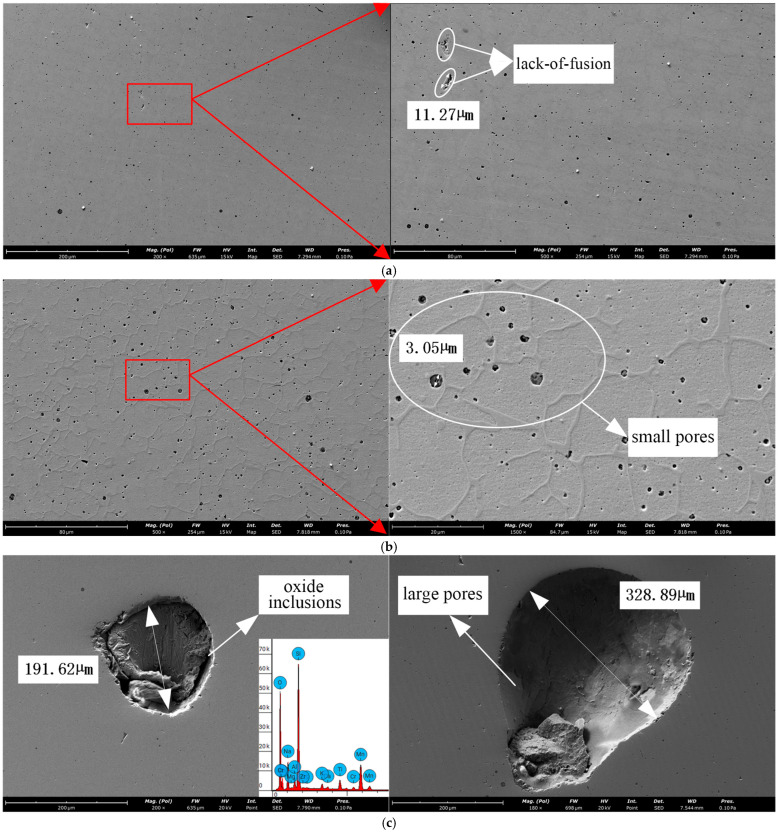
Typical morphological characteristics of internal defects. (**a**) Lack-of-fusion; (**b**) micro-pore, V30; (**c**) oxide inclusion.

As illustrated in Figure 9, the typical defect morphology characteristics within the formed part can be categorized into three types: namely, pores, oxide inclusions, and lack-of-fusion defects. Pores are distributed uniformly across the cross-section, with micro-pores (ranging from 1 to 4 µm in diameter) predominantly present, while larger pores (reaching 300–500 µm) are randomly distributed within the material. During the forming process, a large volume of metal wire is continuously fed into the molten pool. Contaminants such as moisture, grease, and hydrocarbons on the wire surface are vaporized under the high-temperature arc, converting into atomic hydrogen and subsequently being absorbed into the molten pool. Additionally, improper shielding gas output can lead to water molecules in the air dissociating into hydrogen ions, which are also absorbed into the molten pool. It is important to note that the solubility of gases in the molten pool increases with temperature. Due to the rapid melting and solidification of the molten pool, the gases do not have sufficient time to escape entirely during solidification, resulting in the formation of pores. Furthermore, the solidification cavity can also contribute to pore formation.

Lack-of-fusion defects in arc additive-manufactured 316L materials typically manifest as irregular shapes with sharp edges and corners, ranging in size from approximately 11 to 15 µm. These defects are comparatively rare and small in size within the material. The formation of these defects can be attributed to two primary factors. Firstly, insufficient local energy input can result in the upper layer remaining partially unmelted before being covered by the molten pool. Secondly, residual oxide scale on the upper layer edges can hinder the filling of local gaps. Energy-dispersive X-ray spectroscopy (EDS) analysis revealed the presence of large circular defects, with a diameter of approximately 200 µm, which are oxide inclusions. These oxide inclusions are primarily attributable to two sources: firstly, occasional inclusions are caused by improper process control or operations, such as uncrushed slag particles, large splattered slag fragments, or unmelted flux, with sizes reaching the millimeter scale. Secondly, during the crystallization of the molten pool, slag is subjected to crystallization pressure, causing it to diffuse in the direction of grain growth and become embedded within the crystals, resulting in microscopic inclusions.

**Figure 10:**
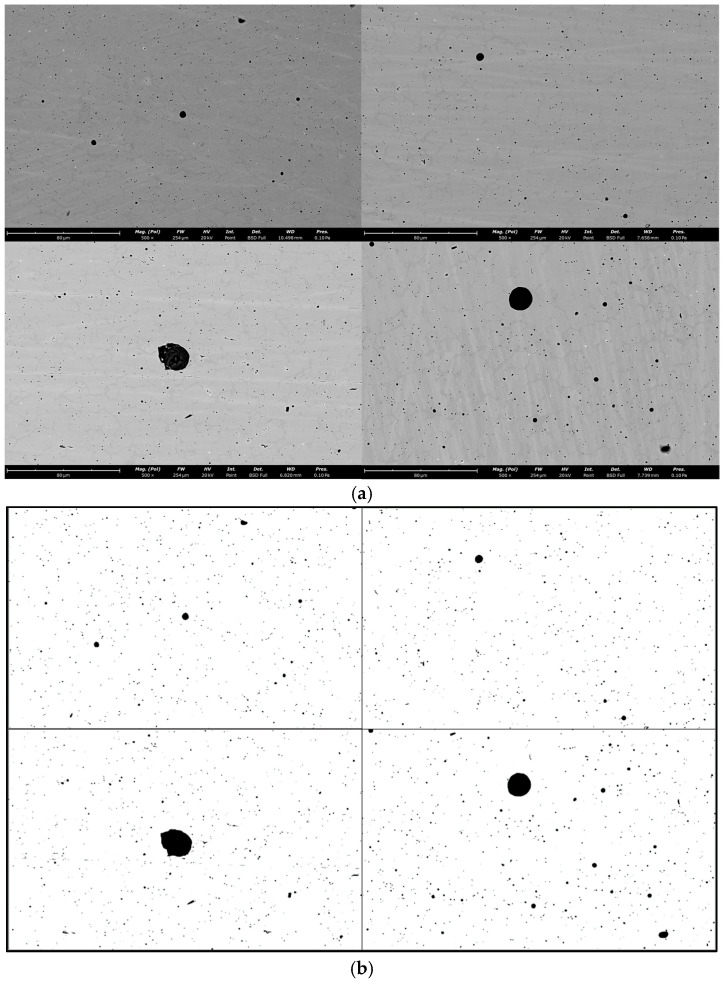
The microstructures of selected WAAM 316L specimens. (**a**) Original micrograph; (**b**) Micrograph after binarization.

In order to observe the number, size, and distribution of defects in the specimens more specifically, two polished specimens with each of the four process parameters (V20, V30, V40, and V20(CO_2_)) were selected for SEM observation in this study. The surface of each specimen was evenly divided into 10 regions, and one random micrograph was captured from each region. Because the vast majority of defects within the specimens are typically small in size, defect characteristics were analyzed at a magnification of 500×. The microstructures of selected WAAM specimens are shown in Figure 10a. The microscopic images of the WAAM 316L specimens were binarized using ImageJ (https://imagej.net/ij/) to distinguish the defects within the material from the matrix. The results after binarization are shown in Figure 10b.

**Figure 11:**
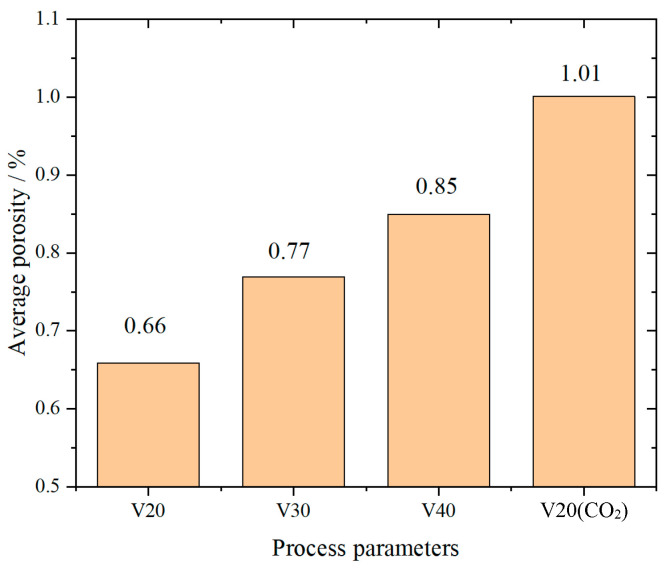
Average porosity under different process parameters.

Porosity, defined as the ratio of pore volume to total volume, is a critical parameter reflecting the density of a material. It is the primary forming defect in arc additive manufacturing materials, and excessive porosity can lead to the deterioration of various mechanical properties, such as strength, toughness, and hardness, thereby compromising the reliability and safety of the manufactured components. Therefore, controlling porosity is essential for achieving high-quality forming in arc additive manufacturing. In this study, the planar porosity was determined by calculating the ratio of the defect area to the total area in microscopic images. Using the binary images of micro-defects derived from Figure 10, the porosity in the forming direction was calculated as the ratio of pixels with a grayscale value of 255 (black regions) to the total number of pixels in the image. Indeed, there is strong distribution on pores by the process. However, upon removing the surface of the plate, it can be observed that the porosity distribution at other locations is relatively uniform. Therefore, we refer to the standard ISO 4967: 2013 [21]. Porosity parameters were analyzed and averaged in 20 random fields of view from multiple specimens to minimize the randomness and dispersion of the calculated results. The porosity values of the specimens formed under different process parameters are presented in Figure 11.

**Figure 12:**
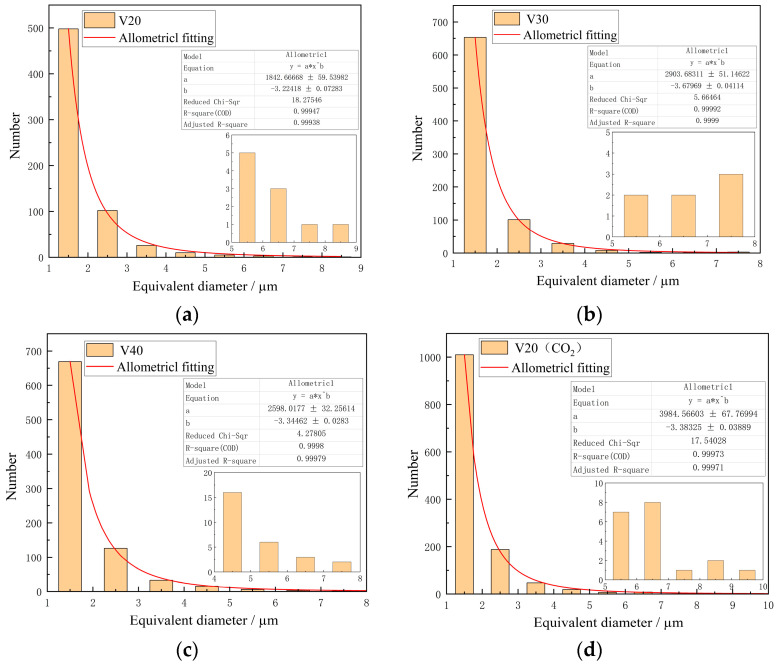
Equivalent diameter under different process parameters. (**a**) V20; (**b**) V30; (**c**) V40; (**d**) V20(CO_2_).

An analysis of the figures indicates a positive correlation between porosity and scanning speed. At higher speeds, the reduced time available for complete liquefaction of the molten wire results in inadequate material densification upon cooling, leading to an increase in porosity. A notable rise in porosity was also observed when the shielding gas was switched. At a constant welding speed, the porosity observed under the V20(CO_2_) parameter was found to be considerably higher in comparison to that observed under the V20 parameter. The defect data were extracted from 20 fields of view for each parameter set using ImageJ, with a particular focus on defects with an equivalent diameter greater than 1 µm. The distribution of defect equivalent diameters, as demonstrated in Figure 12, indicates that for all process parameters, the majority of defects were concentrated in the 1–4 µm range, with small-sized defects being predominant. Additionally, the number of micro-defects (~1.5 µm) increased with scanning speed. Subsequently, the equivalent diameters of the pores generated during the arc additive manufacturing process were subjected to nonlinear fitting based on the allometric model. The fitting results demonstrated a high degree of accuracy, with a determination coefficient greater than 0.99, thus confirming that the model effectively described the distribution of pore diameters.

**Figure 13:**
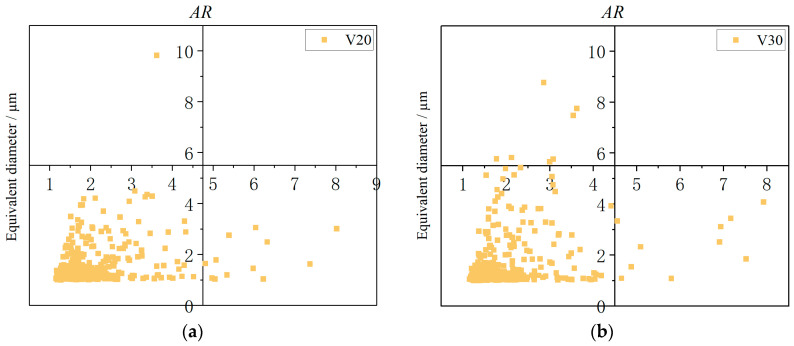

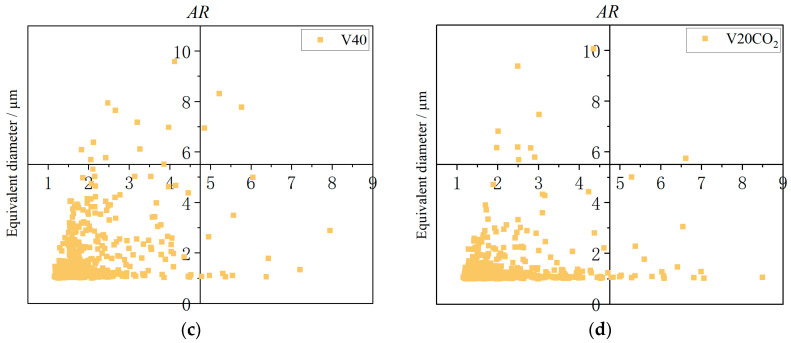
Relationship between equivalent diameter and *AR* under different process parameters. (**a**) V20; (**b**) V30; (**c**) V40; (**d**) V20(CO_2_).

In order to further characterize the morphological features of the defects, the aspect ratio (*AR*) of the defects observed in the SEM micrographs was subjected to analysis. ’Equivalent diameter’ refers to the maximum diameter of each pore, and ‘A*R*’ refers to the ratio of maximum and minimum inner diameters of the pores, given that the majority of the pores are elliptical, with a close proximity to the circle. These data were obtained from the ImageJ software. As shown in Figure 13, the *AR* values of the defects at different scanning speeds are predominantly concentrated between 1 and 2, indicating a relatively high degree of circularity. However, some defects exhibit *AR* values greater than 2, thereby displaying a flattened pore morphology. It is evident that the extent of flattening of the defect is directly proportional to the magnitude of the *AR* value.

### 3.2. Reliability Validation of Nonlinear Ultrasonic Detection Systems

#### 3.2.1. Output Level

In order to validate the effectiveness of the nonlinear ultrasonic testing system, the V20 specimen was selected as the research subject to analyze the influence of output signals at different energy levels on the ultrasonic nonlinear parameter. The energy level range was adjusted from 30 to 65. A 20-cycle Hanning-modulated ultrasonic signal with an excitation frequency of 2.1 MHz was used for excitation, and the detection distance was set to 30 mm. The receiving signal gain was set to 42 dB, and glycerin was used as the coupling agent. For each energy level, three repeated experiments were conducted. The values of *A*_1_, *A*_2_, and the nonlinear parameter *β* are recorded in Table 4.

**Figure 14:**
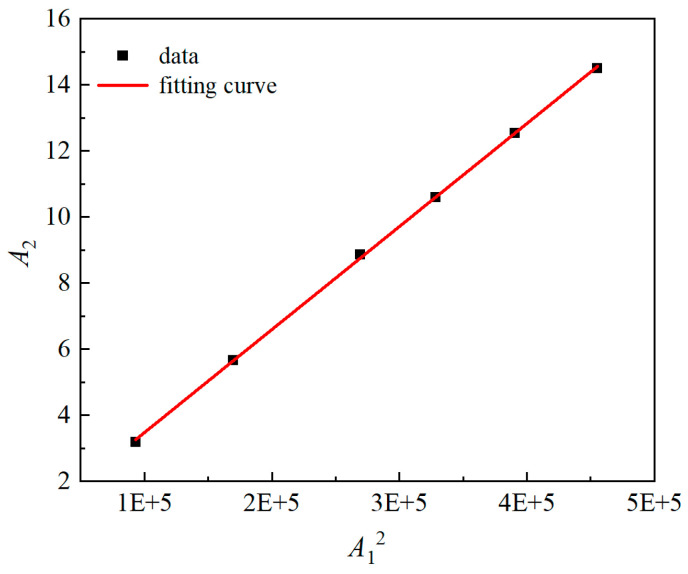
Relationship between $A_{1}^{2}$ and *A*_2_ at different output levels.

Based on the data in Table 3, the relationship curve between $A_{1}^{2}$ and *A*_2_ was plotted. The slope of the curve represents the ultrasonic nonlinear parameter *β*. As shown in Figure 14, a strong linear relationship is observed between $A_{1}^{2}$ and *A*_2_. Furthermore, the ultrasonic nonlinear parameter demonstrates minimal fluctuation under varying output energy levels, thereby indicating that the testing system exhibits excellent stability.

#### 3.2.2. Excitation Frequency

**Figure 15:**
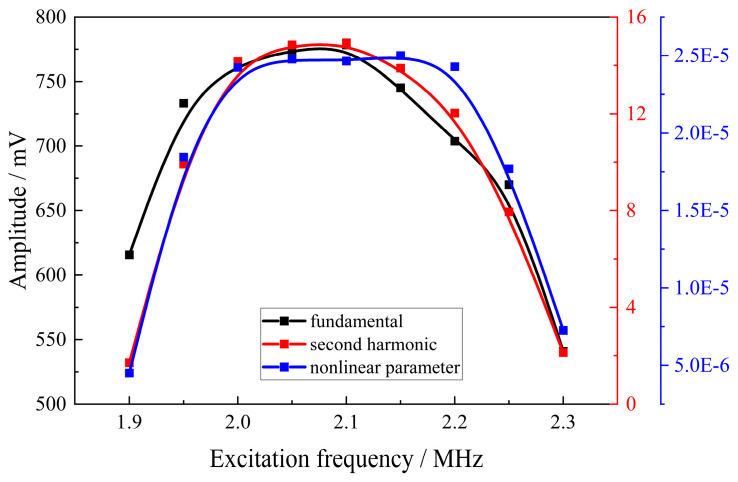
Relationship between nonlinear parameter and excitation frequency.

Nonlinear ultrasonic testing was performed on the V20 specimen under different excitation frequencies, while maintaining constant conditions otherwise. The effect of varying excitation frequencies on the ultrasonic nonlinear parameter was analyzed, with the excitation frequency ranging from 1.9 MHz to 2.3 MHz at intervals of 0.05 MHz. The experimental results are presented in Figure 15. As demonstrated in the figure, the second harmonic amplitude is significantly smaller compared to the fundamental amplitude. The fundamental amplitude, second harmonic amplitude, and relative nonlinear parameter exhibited an initial increase and subsequent decrease with increasing excitation frequency, reaching a peak at an excitation frequency of 2.1 MHz. This frequency was selected to achieve a more prominent second harmonic signal and a higher relative ultrasonic nonlinear parameter.

#### 3.2.3. Propagation Distance

**Figure 16:**
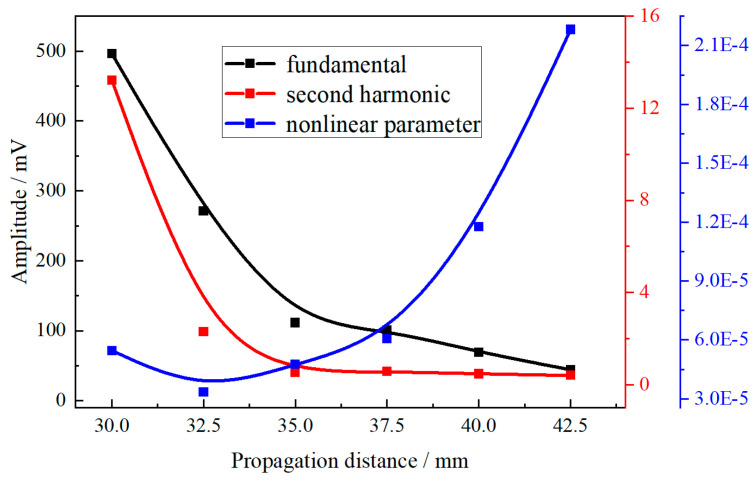
Relationship between nonlinear parameter and propagation distance.

It is expected that, when the output energy of the detection system remains constant, a monotonically increasing relationship between *A*_2_/*A*_1_^2^ and the propagation distance will be observed. This ensures that the second harmonic in the received signal originates from the intrinsic nonlinearity of the specimen rather than the system’s nonlinearity. In this experiment, the output energy level was kept constant, and the detection distance was gradually varied. The excitation signal comprised a 20-cycle Hanning-modulated ultrasonic wave with a frequency of 2.1 MHz. The variation range for the excitation and receiving probes was 30–42.5 mm, with an interval of 2.5 mm. Figure 16 illustrates the relationship between the relative nonlinear parameter and propagation distance. The figure demonstrates that both the fundamental and second harmonic amplitudes decrease with increasing propagation distance, while the ultrasonic nonlinear parameter exhibits an increasing trend, thereby confirming the cumulative effect of ultrasonic nonlinearity. To achieve a distinct second harmonic signal, a propagation distance of 30 mm was selected for subsequent experiments.

### 3.3. Relationship Between Porosity and Ultrasonic Nonlinear Parameters

**Figure 17:**
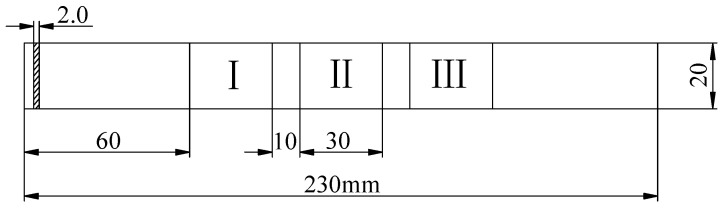
Dimensions of the detection area.

Nonlinear ultrasonic testing was conducted to evaluate the forming defects of the specimens and to investigate the correlation between porosity and ultrasonic nonlinear parameters for specimens formed under different process parameters. The aim was to determine whether nonlinear ultrasonic testing can be utilized to assess the degree of microstructural defects in specimens under specific process conditions. For each process parameter, three specimens were detected using nonlinear ultrasonic techniques. Each specimen was divided into three areas, with each area detected three times, resulting in nine measurements per specimen. A total of 27 measurements were carried out for each process parameter, ensuring that a sufficiently large area was evaluated and simultaneously improving the accuracy and reliability of the data. The division of the detection area is illustrated in Figure 17.

**Figure 18:**
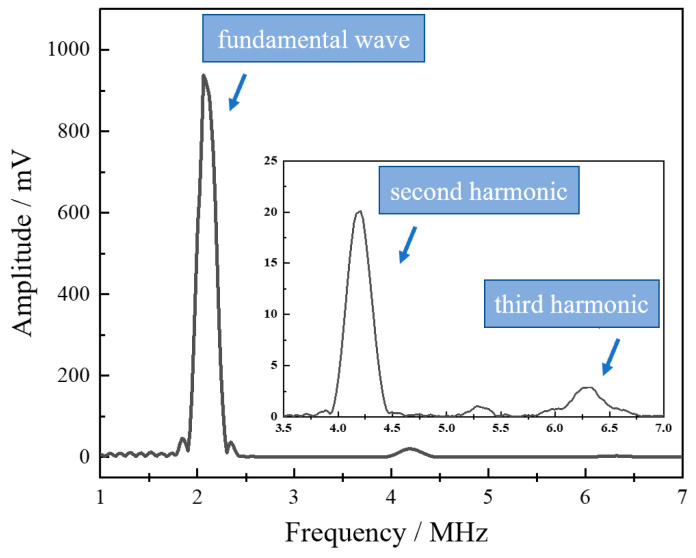
Frequency-domain signal.

Nonlinear ultrasonic testing was carried out on WAAM 316L specimens under four process parameters to investigate the relationship between ultrasonic nonlinear parameters and porosity. It can be seen from the received time-domain signals that obvious second-harmonic signals can be observed, but significant third-harmonic signals also appeared (as shown in Figure 18). Consequently, both second-order and third-order ultrasonic nonlinear parameters were calculated to characterize the internal defects of specimens under different process parameters. The average values of 27 nonlinear ultrasonic tests for each process parameter are summarized in Table 5.

**Figure 19:**
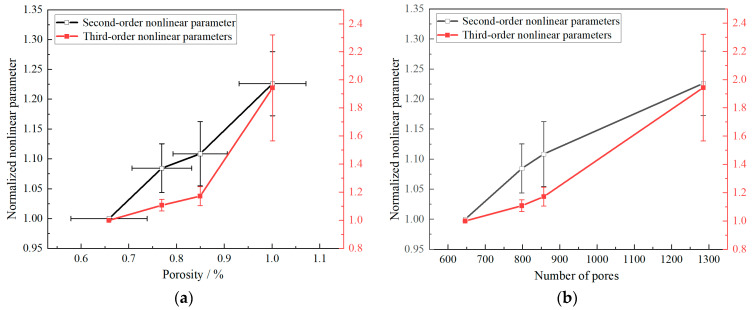
Relationship between defects characterization and normalized nonlinear parameter. (**a**) Porosity; (**b**) number of pores.

The second-order and third-order nonlinear parameters calculated for all process parameters were normalized by dividing them by the corresponding nonlinear parameters under the V20 process condition, yielding normalized relative nonlinear parameters. The relationships between the normalized second-order and third-order nonlinear parameters and porosity are shown in Figure 19. As depicted, the nonlinear parameters increase with rising porosity. Specifically, as the porosity increases from 0.66% to 1.01%, the second-order nonlinear parameter shows a 22.6% increase, while the third-order nonlinear parameter increases by 94.2%. These results indicate that nonlinear parameters can effectively characterize the porosity of WAAM 316L stainless steel. Moreover, compared to the second-order nonlinear parameter, the third-order nonlinear parameter exhibits higher sensitivity to porosity.

Furthermore, the total number of pores (aggregated from all defects within the microscopic field of view) was subjected to analysis for each process parameter. We also used micro-CT scanning to obtain the 3D reconstructed morphology of the specimen, which enabled us to obtain a larger range of more accurate defect characterization parameters. However, due to the size of the specimen and the micro-CT resolution, micro-defects below 3 µm could not be recognized. Consequently, SEM was used in this study to obtain the 2D characteristic parameters of the defects. The relationships between the normalized second-order and third-order nonlinear parameters and the number of pores are illustrated in Figure 19. The nonlinear parameters increased with the number of pores, consistent with their relationship to porosity. This suggests that the number of pores directly determines the porosity, and both exhibit a positive correlation with the ultrasonic nonlinear parameters. In summary, it can be concluded that the nonlinear parameter effectively reflects the porosity and microscopic damage state within the material. Nonlinear ultrasonic testing can be employed to characterize and evaluate the processing defect characteristics and initial damage states of WAAM 316L and other additive manufacturing materials.

## 4. Conclusions

This study systematically investigated the microstructural and defect characteristics of WAAM 316L stainless steel under various process parameters and established the relationship between ultrasonic nonlinear parameters and defect characteristics, such as porosity. The main conclusions are as follows:

(1) The microstructures were analyzed in both the horizontal and vertical directions. It was thus observed that the horizontal cross-section exhibited an equiaxed grain structure, while the vertical cross-section displayed a typical columnar grain morphology. With an increase in the number of deposited layers, there was an expansion of the columnar grain spacing, accompanied by the formation of secondary dendrites.

(2) An analysis of the processing defects under different process parameters was conducted. The results indicated that small pores (1–4 µm) were uniformly distributed throughout the material and dominate the defect population. As the scanning speed increased, the porosity of the specimens showed a rising trend. Furthermore, the porosity exhibited by the shielding gas CO_2_ was significantly higher than that of 98% Ar + 2% O_2_.

(3) Ultrasonic nonlinear parameters exhibited a high sensitivity to micro-defects within WAAM 316L and increased significantly with the rising porosity. It is concluded that ultrasonic nonlinear parameters can be used to characterize the internal porosity of specimens. A notable finding is that, in addition to the second-order nonlinear parameter, the third-order nonlinear parameter also demonstrates high sensitivity to the porosity of WAAM 316L.

## Figures and Tables

**Table 1:** Chemical composition (wt.%) of 316L stainless steel flux-cored wire.

C	Mn	Si	Ni	Cr	Mo	S	P	Cu	Fe
≤0.04	1.3	≤1.0	12.0	18.65	2.42	≤0.03	≤0.04	≤0.75	Remaining

**Table 2:** Main process parameters for WAAM.

No.	Scanning Speed (cm/min)	Shielding Gas
V20	20	98%Ar + 2%O_2_
V30	30	98%Ar + 2%O_2_
V40	40	98%Ar + 2%O_2_
V20(CO_2_)	20	100%CO_2_

**Table 3:** Matrix and precipitate phases of V20 specimen by EDS.

	Element	Atomic Percentage/%	Weight Percentage/%
Precipitate	Si	1.575	0.799
	Cr	27.751	26.074
	Mn	1.107	1.099
	Fe	61.971	62.537
	Ni	5.464	5.794
	Mo	2.132	3.696
Matrix	Si	0.989	0.501
	Cr	21.268	19.920
	Mn	1.416	1.401
	Fe	62.690	63.063
	Ni	12.594	13.313
	Mo	1.043	1.802

**Table 4:** Nonlinear ultrasonic detection results of specimens at different output levels.

Output Level (%)	30	40	50	55	60	65
*A*_1_ (mV)	305.02	411.91	518.68	572.85	624.97	674.87
*A*_2_ (mV)	3.19	5.66	8.86	10.60	12.53	14.50
*β*	3.42 E-5	3.34 E-5	3.29 E-5	3.23 E-5	3.21 E-5	3.18 E-5

**Table 5:** Nonlinear ultrasonic detection results of specimens under different process parameters.

No.	Second-Order Nonlinear Parameter *β*	Third-Order Nonlinear Parameter *δ*	Porosity/%	Number of Pores
V20	2.2806 E-5	3.39 E-9	0.65865	646
V30	2.4731 E-5	3.76 E-9	0.7692	798
V40	2.528 E-5	3.97 E-9	0.8495	857
V20(CO_2_)	2.7961 E-5	6.59 E-9	1.00105	1285

## Data Availability

The original contributions presented in the study are included in the article, further inquiries can be directed to the corresponding author.
